# Key components in models of community-based interventions coordinating care in dementia: a mixed studies systematic review protocol

**DOI:** 10.1186/s13643-015-0143-y

**Published:** 2015-11-07

**Authors:** Amy Backhouse, Chris Dickens, David Richards, Rose McCabe

**Affiliations:** University of Exeter Medical School, College House, St Luke’s Campus, Exeter, EX1 2LU UK; National Institute for Health Research (NIHR) Collaboration for Leadership in Applied Health Research and Care (CLAHRC) South West Peninsula, South Cloisters, St Luke’s Campus, Exeter, EX1 2LU UK

**Keywords:** Systematic review protocol, Mixed studies review, Synthesis, Dementia, Care management, Collaborative care, Case management, Community interventions, Care coordination, Health services

## Abstract

**Background:**

Current health and social care systems are providing suboptimal and fragmented care to the growing dementia population. Interventions aiming to coordinate care services for individuals with dementia and their families are already widely used; however, the structure and implementation of these interventions vary. This mixed studies review aims to investigate the key components of effective community-based interventions that focus on coordinating care in dementia.

**Methods:**

We will search MEDLINE, Cochrane Library, Embase and PsycINFO databases for studies of any design that look at community-based interventions that aim to coordinate dementia care through the allocation of a specified professional responsible for provision of care. Health Management Information Consortium (HMIC), Social Policy and Practice (SPP), ProQuest and International Clinical Trials Registry Platform (ICTRP) databases will be searched for grey literature. Outcomes of interest are health outcome measures that relate to the individual with dementia and/or informal caregiver, measures of resource use or process measures. Two independent reviewers will screen identified papers and extract data from eligible studies. Evidence synthesis will take place in three stages, and methods will be largely dependent on the data available. A sequential review design will be used where the qualitative evidence will be synthesised first, focusing on stakeholder’s subjective views of key components. This will drive forward the quantitative stage which will identify key components of effective interventions. The final stage of the review will merge the two strands of evidence through a narrative synthesis.

**Discussion:**

The results from this review will be used to develop a model for a community-based intervention coordinating care in dementia. Furthermore, the findings will help guide future work on intervention development of health and social care services for dementia.

**Systematic review registration:**

PROSPERO CRD42015024618

**Electronic supplementary material:**

The online version of this article (doi:10.1186/s13643-015-0143-y) contains supplementary material, which is available to authorized users.

## Background

### Rationale

Dementia is fast becoming one of the greatest challenges to health and social care services in the UK. With an ageing population, the prevalence of dementia is growing rapidly with current UK estimates of 800,000 individuals living with dementia which is predicted to rise to over one million by 2021 [1]. Alongside the increasing prevalence come substantial costs. A recent report from the Alzheimer’s Society states that the full societal cost of dementia has now reached an annual total of £26.3 billion, a figure comprising of costs for direct healthcare, social care (both private and publically funded) and the informal carers of people with dementia. This figure is greater than the combined costs of heart disease and stroke [2].

The complexity of needs that distinguishes this population must be addressed through a holistic and comprehensive approach to care. However, a number of recent government reports [3] and clinical guidance documents [4] have described the varying components of healthcare and social care responsible for the provision of dementia care as fragmented and poorly coordinated. These reports highlight the discrepancies between what current services are providing for the dementia population and what the research evidence suggests is effective high quality care, concluding that available services are suboptimal in standards and offer poor value for money. There is a frequent failure in individuals accessing recommended services and a lack in systematic help finding these services. Furthermore, when these services are accessed, there is an absence of continuity in long-term support [1], all of which leave capacity to improve the quality, efficiency and costs of dementia care.

One approach taken to improve the management of care provided to this population is the allocation of a health or social care professional to assume the responsibility of coordinating all aspects of care for the individual. These are organisational interventions which have been under development over recent decades and have been applied to a number of long-term conditions including dementia. The organisation and implementation of such interventions varies widely, but the primary focus is to develop a collaborative process of planning, facilitating and coordinating care and providing a proactive support base for both the patient and their informal caregiver/families. In the literature, the variations in these interventions have taken a number of different titles which are often used loosely and interchangeably including care/case management, collaborative care and care coordination.

The diversity and complexity present in these models of care has led to a patchy evidence base and a difficulty in defining the key components to measure their success. There have been a number of systematic reviews that have drawn mixed conclusions on the efficacy and impact of coordinating interventions [5–10]. Tam-Tham et al. [9] reported short-term effects of dementia case management on risk of institutionalisation, a result in agreement with Pimouguet et al. [8] who also reported a number of high-quality trials demonstrating a positive impact of dementia case management in delaying institutionalisation. However, neither review found benefits on any further outcome measures. On the contrary, Somme et al. [7] reported on a number of randomised controlled trials (RCTs) demonstrating moderate effects on both clinical outcomes and resource utilisation.

Reviews of this nature face difficulty with the extent of heterogeneity across studies. This heterogeneity comes from a number of aspects including participant characteristics such as demographics, gender and disease severity; intervention characteristics such as case load, intensity and method of delivery; and contextual factors or from variation in outcome measures assessed [11]. With diversity in both the models of care and implementation methods, it becomes difficult to compare the impact of the interventions and what components are essential for its effectiveness. We will attempt to address these issues using a mixed studies review that is designed to be comprehensive, allowing the use of a vast range of research to address the complex nature of such health care interventions. Here, we outline the protocol of our review in accordance with the PRISMA-P [12] (2015) statement for preferred reporting items for systematic review and meta-analysis protocols (see Additional file 1).

### Objectives

The aim of this review will be to identify the key components of community-based interventions coordinating care in dementia by asking the following questions:

- 1. What are the experiences, perceptions and views of stakeholders on community-based interventions coordinating care in dementia in terms of:

(i) the perceived key components of the interventions?
(ii) the helpful/unhelpful characteristics of the interventions?
(iii) the proposed mechanisms by which the interventions achieve their effect?

- 2. What are the effects of community-based interventions coordinating care in dementia on patient and carer outcomes?
- 3. Is there a relationship between the perceived key components of these interventions and the patient and carer outcomes?

To address these review questions, we will conduct an exploratory, sequential review design where the qualitative evidence will be synthesised first and will drive forward the secondary synthesis of quantitative evidence. With such a design, the quantitative results are mobilised to examine, confirm and generalise the qualitative findings [11]. Insights from both strands will be combined during the final stages of the review.

## Methods

### Eligibility criteria

#### Types of studies

We are conducting a mixed studies systematic review, and therefore, we will include studies of any design. We will consider inclusion of, but not limited to, the following study designs:

1. Qualitative study design: qualitative studies that canvass the experiences, perceptions and views of stakeholders on dementia care coordination interventions.
2. Quantitative study design: quantitative studies may include randomised controlled trials (RCTs) of intervention effectiveness or non-RCT designs (e.g. quasi-experimental, controlled/non-controlled before and after, cross-sectional and cohort studies).
3. Mixed methods study design: we will also consider studies that have incorporated both strands in the methodological design.

No restrictions will be placed on study location. However, a flexible approach will be taken to the context of the study during the screening process, with particular care being paid to the global differences in health and social care systems. No restriction will be placed on language; however, a flexible approach will be taken here when considering the context of the study, in particular with regards to qualitative research, where study findings will be context specific and concepts can be lost in translation which may lead to a greater degree of inference. No restrictions will be applied to date of included studies.

#### Types of participants

We will include participants of any age and gender who are living in the community with a dementia diagnosis of any type (made by a clinician, such as a general practitioner (GP) or a hospital specialist, or made following standardised neuropsychiatric assessment) and their informal caregiver (if data are available). We will exclude studies that include people who self-define as having dementia (i.e. in the absence of a formal clinical diagnosis). The diagnostic uncertainty surrounding such participants is likely to increase the heterogeneity of our findings, and would definitely reduce the confidence and credibility with which we would be able to make future treatment recommendations for people with dementia, which is the main aim of our review. We will exclude studies that focus solely on interventions for the informal caregiver, which do not include an intervention focused on increased coordination of care or outcomes for the individual with dementia. Studies that do not focus on improving coordination of dementia care are beyond the scope of this review.

#### Types of intervention

We will include studies of interventions that are delivered in community settings to the specified population. The intervention must have an identified professional, key in the provision and management of care, who has a primary focus on care planning, coordination and proactive follow-up tailored to the needs of individual with dementia and/or informal caregivers. We will exclude any non-community-based interventions such as those based in hospitals, care or residential homes. We will exclude studies that involve changes made to health care systems or application of guidelines alone.

#### Comparator

Many of the studies included will be of a qualitative or non-controlled design and therefore will not comprise a comparator group. Some quantitative studies reaching inclusion criteria, such as RCTs, will involve case-acceptable comparators such as treatment as usual, placebo control, waiting-list control or alternative dementia care interventions.

#### Types of outcome measures

We will examine a range of primary and secondary outcome measures. We will include studies that report on one or more health outcome measures that relate to the individual with dementia and/or informal caregiver, measures of resource use or process measures. These will have used standardised and validated measurement tools. Health outcome measures may include, but are not limited to, the following: depression and anxiety, carer burden, quality of life and cognition measures. Measures of resource use may include, but are not limited to, the following: institutionalisation, hospital admission rate and mean length of stay. Process measures may include, but are not limited to, the following: frequency of contact, case load intensity and professional background of case managers.

### Information sources

- (1) Database search: Electronic databases will be searched from data of inception to present, and the search syntax will be modified as appropriate for use in the following databases:

- MEDLINE (OvidSP)
- The Cochrane Library
- EMBASE
- PsycINFO

- (2) Citation search: Forward and backward citation searching will be conducted on included articles for further material. In the absence of required information, the first authors of studies will be contacted to request additional related material either unpublished or in press.
- (3) Experts in the field: Experts in the field and corresponding authors of included studies will be contacted to gather further information.
- (4) Grey literature search: To minimise the impact of publication bias, grey literature sources will be searched for unpublished material. Despite previous variations in the definition of grey literature, it has become commonly known as material that is not controlled by commercial publishers [13]. Examples of this type of material include government reports, policy documents, dissertation theses, book chapters and research reports. Searches for grey literature will be conducted in the Health Management Information Consortium (HMIC) and the Social Policy and Practice (SPP) databases, both of which will be accessed via OvidSP. HMIC is house to material including, but not limited to, Department of Health (DH) reports, Kings Fund data, and relevant health care books. SPP holds material including, but not limited to, government reports, health and social care services information and policy documents. The ProQuest database will be searched specifically for dissertations and theses. For additional information and material regarding RCTs, the International Clinical Trials Registry Platform (ICTRP) search portal will be used with an adjusted search strategy specified to the tools available on this platform.

### Search strategy

A comprehensive search strategy has been developed and will use both controlled vocabulary unique to each database (e.g. MEDLINE Medical Subject Headings (MeSH) terms) and free-term texts. The strategy has been informed by discussions with experts in the field of dementia, complex interventions, systematic reviewing and by a prior scoping review identifying relevant keywords. An outline of the master search strategy for MEDLINE (OvidSP) can be found in Appendix 1. Our choice of electronic databases includes sources of grey literature to minimise the effects of publication bias. In addition, we will conduct a forward and backward citation searching of relevant papers and contact experts in the field for details of unpublished studies.

### Study records

#### Data management

EndNote X7.0.2 software will be used to manage references throughout the review. Once the searches have been run, results will be exported to EndNote and any duplicates automatically identified will be removed. This process will be assisted by hand searching for duplicates.

#### Screening

Two independent reviewers will conduct an initial screening of titles and abstracts of identified papers for relevance in accordance with the outlined eligibility criteria. Full texts of potentially eligible studies will be retrieved, and a second screening will be conducted by two independent reviewers to produce a final set of papers to be included in the review. Disagreement at any stage will be resolved through discussion and referral to a third reviewer. A PRISMA diagram will be completed to show the flow of the screening process and number of records at each stage.

#### Data extraction

A bespoke data extraction sheet that has been developed in-house will be used. We anticipate that these will primarily be data-driven by studies found, but will be based on standardised forms for quantitative and qualitative extraction, and will be tailored to the characteristics of this review. The data extraction sheets will be piloted on six studies (three quantitative and three qualitative) identified at random from eligible studies and modified as required for use in this review. Once the final set of included studies has been established, data extraction sheets will be completed by two independent reviewers.

Data items to be extracted from qualitative studies will include, but not limited to, the following:

- General information (author(s), title, year of publication, journal of publication, country, and language).
- Study design (setting, recruitment, sample size, data collection, analysis).
- Participant characteristics (gender, age, stakeholder group).
- Data—text from ‘results’ and ‘findings’ from research reports. This data is likely to be presented in the form of themes and quotations.

Data items to be extracted from quantitative studies will include, but not limited to, the following:

- General information (author(s), title, year of publication, journal of publication, country, and language).
- Study design (setting, recruitment, eligibility, sample size, randomisation, data collection, analysis).
- Participant characteristics (gender, age, diagnosis, comorbidities).
- Intervention (coordinating role, training, intensity, follow-up period, comparison groups).
- Outcome measures—Primary and secondary outcomes will be extracted from quantitative studies, including health outcome measures for both individual with dementia and caregiver and health process measures. This data is likely to include means, standard deviations and effect sizes.

We anticipate studies that have included mixed methodology will likely undergo two data extractions; although there will be some overlap, data will be extracted for the qualitative data sheet and for the quantitative data sheet. Where multiple publications have been produced from one study, data extraction will be conducted for each individual paper though the data will be considered as one study.

### Risk of bias

Quality appraisal of included studies will be conducted by two independent reviewers using the Critical Appraisal Skills Programme (CASP) checklists [14]. CASP has a set of eight critical appraisal tools, and the tool selected for quality appraisal will be specific to the study design. We will consider the impact of the study quality on the findings of the review, possibly by comparing studies of high versus low quality, if sufficient suitable studies are identified. Assessing the quality of evidence in the review will also allow us to judge the strength of the conclusions and following recommendations.

### Data synthesis

Synthesis of qualitative research will be dependent on the data. Where enough conceptual data is available, then it is anticipated that thematic synthesis, based on methods described by Thomas and Harden [15], will be used to address the initial review question and sub-review questions. In qualitative research, it can become difficult in defining what amounts to data, but here, our primary source of data will be, but not limited to, the text labelled as ‘results’ or ‘findings’ in study reports. Thematic synthesis will take form across three stages, beginning with an initial coding of primary data. Similarities and differences will then be sought within the codes allowing them to be organised into data-driven descriptive themes and permitting translation of concepts between studies. The final stage will involve the generation of analytic themes which will be driven by the review questions and their theoretical framework. However, if interpretive synthesis is not suitable for the data extracted, then a narrative synthesis approach will be taken.

Findings from the quantitative studies will be presented using narrative text and tabulation of the study characteristics, outcomes and risk of bias. Meta-analysis of RCTs will be considered if sufficient trials using comparable interventions and outcomes are identified. Outcomes from trials will be converted to the most appropriate standardised effect, which will then be pooled, using meta-analytic techniques [16]. Variation of study effect with differences in study, participant, setting and intervention characteristics will be estimated using the analogue of analysis of variance (ANOVA) or meta-regression, as appropriate. If meta-analysis is possible, publication bias will be assessed using Egger’s test, forest plots and Duval’s Trim and Fill methods.

Trials will only be considered for inclusion in the meta-analysis if the effects of the coordinating intervention can be isolated from that of the control. Acceptable designs for a trial to be incorporated into the meta-analysis include, for example:

- (1) Coordinating intervention plus treatment as usual versus treatment as usual
- (2) Coordinating intervention plus alternate intervention versus alternate intervention

Trials will not be included in the meta-analysis where effects of target intervention cannot be isolated, for example, target intervention versus alternate intervention. Studies with this later design will be described in the narrative section and tables but will be excluded from meta-analysis.

Once analysis of data is complete, the final stage of the review will merge the two strands of data through a narrative synthesis to conclude the findings and propose how the project will progress to develop a community-based intervention for coordinating care in dementia.

## Discussion

This mixed studies review will provide a detailed account of the evidence base underpinning interventions that coordinate healthcare for people with dementia. Synthesis of study findings will isolate components of interventions perceived as important by identified stakeholders and those that are effective at improving outcomes. Our interpretation of the synthesis of study findings will take account of limitations in studies identified and any limitations in our own review methodology.

To our knowledge, this is the first review to address associations between perspectives of stakeholders and the effectiveness of interventions. The mixed studies sequential design is required for the multiple layers of review questions and allows us to gain both breadth and depth in covering evidence from multiple sources. However, this review design is demanding in time and resources. The use of multiple reviewers will help reduce time burden and minimise the risk of bias. We will discuss how the findings of our review compare and contrast with findings from other reviews of effectiveness of coordinating interventions in dementia [5–11].

The findings of this review will be used to develop a theoretical model of an intervention coordinating dementia care for evaluation in future research. We will also discuss how the evidence supporting care coordinating interventions for people with dementia will be useful to both policy makers and health care providers, informing the future design of services and specific interventions for people with dementia.

## Appendix

### Appendix 1: Master Search Strategy in MEDLINE (OvidSP)

Database: Ovid MEDLINE(R) In-Process & Other Non-Indexed Citations and Ovid MEDLINE(R) <1946 to Present>

Search strategy:

1 exp Dementia/

2 dement*.mp.

3 alzheimer*.mp.

4 (presenile/ or senile.mp.) and dement*.mp. [mp = title, abstract, original title, name of substance word, subject heading word, keyword heading word, protocol supplementary concept word, rare disease supplementary concept word, unique identifier]

5 *Delirium, Dementia, Amnestic, Cognitive Disorders/

6 *cognition disorders/ or *mild cognitive impairment/

7 1 or 2 or 3 or 4 or 5 or 6

8 Case Management/

9 collaborative care.mp.

10 case manag*.ti,ab.

11 care manag*.ti,ab.

12 (care adj2 coordinat*).ti,ab.

13 (case adj2 coordinat*).ti,ab.

14 service coordinat*.ti,ab.

15 care consult*.ti,ab.

16 case consult*.ti,ab.

17 (care adj2 facilitat*).ti,ab.

18 shared care.ti,ab.

19 (coordinat* adj2 care).ti,ab.

20 admiral nursing.mp.

21 *disease management/

22 8 or 9 or 10 or 11 or 12 or 13 or 14 or 15 or 16 or 17 or 18 or 19 or 20 or 21 (33785)

23 7 and 22

***************************

[mp=title, abstract, original title, name of substance word, subject heading word, keyword heading word, protocol supplementary concept word, rare disease supplementary concept word, unique identifier]

[ti,ab=title & abstract]

## Additional file

Additional file 1:
**PRISMA-P 2015 Checklist.** Items that have been reported in the protocol from the required PRISMA-P checklist. (DOC 17 kb)

## Abbreviations

ANOVA: analysis of variance
CASP: Critical Appraisal Skills Programme
DH: Department of Health
GP: general practitioner
HMIC: Health Management Information Consortium
ICTRP: International Clinical Trials Registry Platform
MeSH: Medical Subject Headings
NIHR: National Institute for Health Research
RCT: randomised controlled trial
SPP: Social Policy and Practice
